# Radiofrequency Ablation for Focal Atrial Tachycardia Originating From the Fossa Ovalis: Experiences and Outcomes

**DOI:** 10.1002/clc.70219

**Published:** 2025-11-12

**Authors:** Mingxian Chen, Xuping Li, Zhuo Wang, Jiantong Zhu, Min Zhong, Qiming Liu, Shenghua Zhou

**Affiliations:** ^1^ Department of Cardiology The Second Xiangya Hospital of Central South University Changsha Hunan China; ^2^ Department of Cardiology Wuhan Renmin Hospital of Wuhan University Wuhan Hubei China; ^3^ Department of Cardiology Xiangyang Central Hospital Xiangyang Hubei China; ^4^ Department of Cardiology Huichang County People's Hospital Huichang Ganzhou, Jiangxi China

**Keywords:** atrial tachycardia, bi‐atrial mapping, fossa ovalis, radiofrequency ablation

## Abstract

**Background:**

This study aimed to investigate the electrocardiographic characteristics, electrophysiological features, and outcomes of radiofrequency ablation in patients with focal atrial tachycardia (FAT) originating from the fossa ovalis (FO).

**Methods:**

We retrospectively analyzed 67 patients with FAT originating from the FO and classified into two groups: the Unilateral Ablation Group (*n* = 36) and the Bilateral Ablation Group (*n* = 31). Patients in the Unilateral Ablation Group underwent ablation on the earliest single side, whereas patients in the Bilateral Ablation Group underwent ablation on both the right and left earliest sides. Ablation targets were guided by fluoroscopy, three‐dimensional mapping, and intracardiac ultrasound. All patients were followed up for more than 1 year.

**Results:**

Out of 1914 patients with atrial tachycardia, 3.5% had FAT originating from the FO. Fifty‐four patients were located at the superior area of the FO with positive P waves in inferior leads, while 13 patients were located at the inferior area of the FO with negative P waves in inferior leads. The recurrence rate of FAT was 16.6% in the Unilateral Ablation Group, but no recurrence occurred in the Bilateral Ablation Group during regular follow‐up (*p* = 0.026). Among the six patients with recurrence, five underwent left‐sided ablation and one underwent right‐sided ablation. All recurrent cases were then ablated by a bilateral strategy. Follow‐up showed no further recurrence.

**Conclusions:**

Bi‐atrial mapping is necessary for ablation of FAT arising from the FO. Bilateral ablation for FO AT appears to be more reasonable.

## Graphical abstract

This graphical summary illustrates the electrocardiographic and anatomical features of focal atrial tachycardia (FAT) originating from the fossa ovalis (FO), along with ablation strategies and outcomes. Panel A shows distinct P wave patterns on surface ECG that help differentiate superior from inferior FO origins. Panel B depicts the anatomical distribution of FAT sites in the FO region and highlights the study's classification into superior (*n* = 54) and inferior (*n* = 13) areas. Panel C presents an anatomical view of the FO within the atrial septum. The study demonstrates that bilateral ablation, guided by bi‐atrial mapping, significantly reduces recurrence compared to unilateral ablation in patients with FO‐FAT.

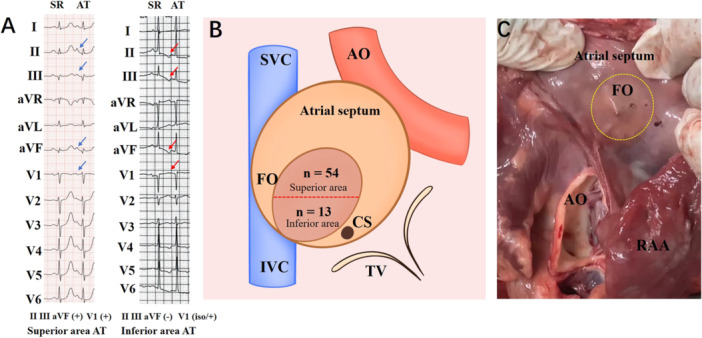

## 1 Introduction

Focal atrial tachycardia (FAT) is a common type of cardiac arrhythmia characterized by rapid and irregular heartbeats originating from specific foci within the atria. While FAT can arise from various atrial regions, including the crista terminalis, tricuspid annulus, coronary sinus (CS), and pulmonary veins [1, 2, 3], cases originating from the fossa ovalis (FO) are rare [4]. The FO, a depression in the interatrial septum, represents a unique anatomical structure with the potential to serve as a focal point for arrhythmogenesis.

Radiofrequency ablation has emerged as a cornerstone treatment for FAT, offering the potential for symptom relief and improved quality of life in affected patients. However, the optimal ablation strategy for FAT originating from the FO remains a subject of debate, with limited data available to guide clinical practice [5, 6]. Bilateral radiofrequency ablation, targeting both the right and left atria near the FO, has been proposed as a potential approach to improve procedural outcomes and reduce arrhythmia recurrence in these patients.

In this study, we aimed to investigate the experiences and outcomes of bilateral radiofrequency ablation for FAT originating from the FO. We sought to assess the electrocardiographic characteristics, electrophysiological features, and long‐term efficacy of this ablation strategy in a cohort of patients treated across multiple centers. By evaluating the feasibility, safety, and effectiveness of bilateral ablation in this specific patient population, we aimed to provide valuable insights into the management of FAT originating from the FO and contribute to the optimization of ablation strategies for this challenging clinical scenario.

## 2 Methods

### 2.1 Study Population

Approval for this study was obtained from the Institutional Review Boards of The Second Xiangya Hospital of Central South University, Wuhan Renmin Hospital of Wuhan University, Xiangyang Central Hospital, and Huichang County People's Hospital. A total of 1914 patients with FAT undergoing radiofrequency catheter ablation (RFCA) were consecutively enrolled between January 2016 and March 2023. Among them, this retrospective analysis focused on 67 consecutive patients with clinically documented FAT originating from the FO.

### 2.2 Electrophysiological Study (EPS)

Before the procedure, each patient provided informed consent and underwent an EPS using the Lead 2000 electrophysiology system (Sichuan Jinjiang Electronic Science and Technology Co. Ltd). Antiarrhythmic drugs were discontinued for at least five half‐lives before EPS. A decapolar catheter was advanced into the CS via the right internal jugular vein, while three quadripolar catheters were positioned at the right ventricular apex, His bundle, and right atrium (RA). Programmed atrial and ventricular stimulation was conducted to assess electrophysiological properties. FAT was diagnosed based on standard electrophysiological criteria and induced using programmed atrial extra stimulation or atrial burst pacing. In cases where clinical atrial tachycardias did not occur spontaneously, intravenous isoproterenol was administered to induce ATs.

### 2.3 Mapping of Atrial Tachycardia

After confirming the diagnosis of focal AT, a three‐dimensional reconstruction of the right chamber of interest was obtained using multipolar mapping catheters the CARTO3 electroanatomical mapping system (Biosense Webster, Diamond Bar, CA, USA), or the Ensite (Abbott Medical). A stable intracardiac electrode on the CS catheter, relative to P‐wave onset, served as a fiducial point for point mapping. Color‐coded local activation time (LAT) maps of the atrium were reconstructed by annotating LAT relative to a catheter positioned into the CS. The origin of the focal AT was defined as the site showing the earliest bipolar electrogram, with a LAT of > 20–30 ms before the onset of the p‐wave. Furthermore, using unipolar recordings, the site of origin was confirmed to have a pure negative (QS morphology) deflection. If the origin of focal AT is confirmed to be from the right‐sided atrial septum, then left chamber was performed with electroanatomical and activation mapping. Transseptal puncture was performed using a BRK1 needle and a long vascular (SL1) sheath. Mapping of the left‐sided FAT originating from FO used a reversed *U*‐curve catheter method for the left‐sided FO after the transseptal puncture. If the patients had a patent foramen ovale, we still performed a transseptal puncture to gain better operation to the left atrium. After bilateral three‐dimensional anatomical reconstruction and activation mapping, the earliest activation sites on both sides were identified, followed by unilateral or bilateral ablation [7].

### 2.4 FO Identification

FO identification involved several steps: First, a long sheath and ablation catheter were guided into the SVC under fluoroscopic guidance. As they were withdrawn simultaneously, the sheath tip jumped twice. After the second jump, the ablation catheter tip was directed towards the FO area. Second, anatomical mapping of the right side was conducted for each patient. The low‐voltage area in the atrial septum was meticulously marked, with this zone considered indicative of the FO area. Thirdly, local electrograms at the FO typically exhibited a double potential in sinus rhythm (SR), comprising a far‐field component from the left atrium and a near‐field component from the right‐sided septum. During AT, this double potential often demonstrated characteristic morphological changes (increased sharpening and/or widening of component deflections). Sites exhibiting these dynamic changes were prioritized as ablation targets. Lastly, intracardiac echocardiography (ICE) was employed to directly identify the right or left FO. ICE was used in selected patients to facilitate anatomical visualization when necessary, such as in difficult transseptal puncture cases. Otherwise, standard fluoroscopic and 3D mapping guidance were employed for all procedures.

### 2.5 Radiofrequency

RF ablation was performed using irrigated ablation catheters (THERMOCOOL SMARTOUCH Catheter or TactiCath Contact Force Ablation Catheter, Sensor Enabled), depending on the operator's discretion. Continuous RFCA with a power of 30 W, a temperature limit of 43°C, and an irrigation rate of 17 mL/min was applied at the right and left‐sided FO. Left‐sided ablation was performed on patients using the reversed *U*‐curve catheter method under fluoroscopic guidance. Patients in the Unilateral Ablation Group underwent ablation at the single earliest site, while patients in the Bilateral Ablation Group underwent ablation at both the right and left earliest sites. AT readily terminated and converted into SR during ablation. An additional 3 min of ablation was performed at the focus area. At the end of the procedure, no further AT was inducible. Intracardiac ultrasound was utilized to further assess the contact between the ablation catheter and the target site on FO. Programmed stimulation was repeated half an hour after ablation. Acute procedural success was defined as the absence of tachycardia or ectopy 30 min following ablation, even after administering isoproterenol infusion (up to 6 μg/min) and performing burst atrial pacing. Following the procedure, patients were monitored for 24−48 h before discharge.

### 2.6 Follow‐Up

All patients were followed up after the last ablation procedure. Information regarding symptoms, the continuation of antiarrhythmic drugs, and the occurrence of arrhythmia recurrence was systematically collected. Follow‐up was confirmed using 24‐h Holter monitor recordings by their referring cardiologists every 3−6 months for the first year and annually thereafter. Arrhythmia recurrence was defined as focal AT lasting 30 s or more during clinical follow‐up. Two clinicians, who were not involved in the ablation procedures, collected and reviewed the data to ensure completeness and accuracy. All patients were followed up for more than 1 year. All patients after the RF ablation procedure received one aspirin (100 mg) tablet daily for 1 month.

### 2.7 Statistical Analysis

SPSS software version 22.0 (SPSS Inc., Chicago, IL, USA) was used for data analyses. Continuous variables were presented as means ± standard deviations and compared using independent samples *t*‐tests. Categorical variables were presented as frequencies and percentages and analyzed using Fisher's exact tests. Non‐normal variables were expressed as median (1st–3rd quartile). The Wilcoxon rank‐sum test for non‐normally distributed variables. Event‐free survival was estimated by the Kaplan–Meier method; we compared the patients between the Unilateral Ablation Group and the Bilateral Ablation Group using the log‐rank test. A *p* value less than 0.05 was considered statistically significant.

## 3 Results

### 3.1 Anatomic Location of AT

**Figure 1:**
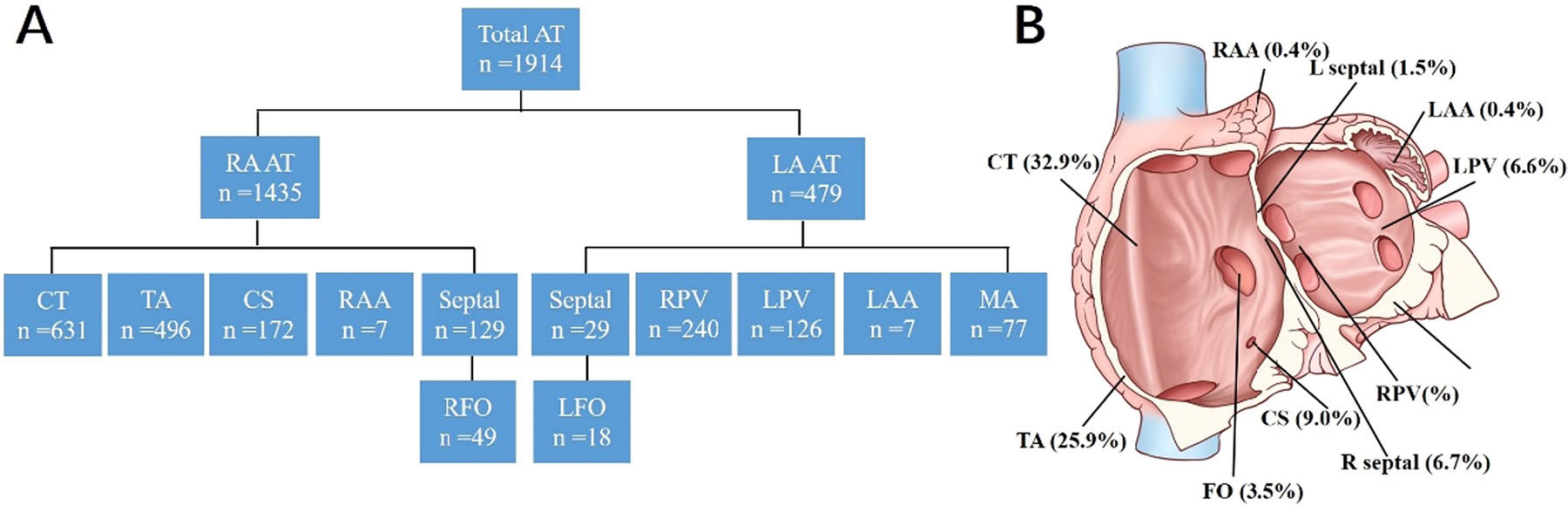
A schematic representation of the anatomical distribution of FAT. The atrioventricular valvular annuli have been removed. AT = atrial tachycardia, CS = coronary sinus, CT = crista terminalis, LA = left atrium, LAA = left atrial appendage, LFO = left‐sided fossa ovalis, MA = mitral annulus, PV = pulmonary vein, RA = right atrium, RAA = right atrial appendage, RFO = right‐sided fossa ovalis, TA = tricuspid annulus.

We collected data from 1,914 patients with FAT. The anatomical distribution of the 1914 AT foci is presented in Figure 1. Among them, 1435 of 1914 (75.0%) were located in the RA, while 479 of 1914 (25.0%) were in the left atrium. Right atrial sites included the crista (*n* = 631), tricuspid annulus (*n* = 496), CS ostium (*n* = 172), perinodal region (*n* = 7), right septum (*n* = 129), and right atrial (RA) appendage (*n* = 7). Left atrial (LA) sites included the pulmonary veins (*n* = 366), mitral annulus (*n* = 77), left septum (*n* = 29), and LA appendage (*n* = 7).

### 3.2 Baseline Characteristics Between Groups

Based on the provided Table 1, the results of the comparison between the Unilateral Ablation Group (*n* = 36) and the Bilateral Ablation Group (*n* = 31) groups are as follows Table 1. The difference was not statistically significant in age, sex, hypertension, diabetes mellitus, coronary heart disease, dyslipidemia, structural heart disease, and ultrasound results between the Unilateral Ablation Group and the Bilateral Ablation Group. The percentage of patients with another arrhythmia was 13.9% in the Unilateral Ablation Group and 9.7% in the Bilateral Ablation Group. There was no significant difference between the groups (*p* = 0.716).

**Table 1:** Baseline Characteristics of FAT originating from FO between two groups.

Variables	Unilateral ablation group (*n* = 36)	Bilateral ablation group (*n* = 31)	*p*
Age (years)	55.7 ± 10.2	56.7 ± 8.9	0.818
Male, *n* (%)	11 (30.6%)	7 (22.6.0%)	0.583
Hypertension, *n* (%)	9 (25.0%)	6 (19.4%)	0.770
Diabetes mellitus, *n* (%)	3 (8.3%)	1 (3.2%)	0.618
CAD, *n* (%)	2 (5.6%)	2 (6.5%)	1.000
Dyslipidemia, *n* (%)	11 (30.6%)	9 (29%)	1.000
Structural heart disease, *n* (%)	1 (2.8%)	1 (3.2%)	1.000
Another arrhythmia (AVNRT/Ventricular ectopy), *n* (%)	5 (13.9%)	3 (9.7%)	0.716
LVEF (%)	62.3 ± 8.2	61.3 ± 5.5	0.545
RA diameter	30.5 ± 2.6	29.7 ± 2.1	0.159
LA diameter	29.6 ± 2.9	29.9 ± 2.6	0.738

*Note:* Values are mean ± SD or *n* (%).

Abbreviations: AVNRT = atrioventricular nodal re‐entry tachycardia, CAD = coronary artery disease, LA = left atrium, LVEF = left ventricular ejection fraction, RA = right atrium.

### 3.3 P‐Wave Morphology (PWM) During AT

**Figure 2:**
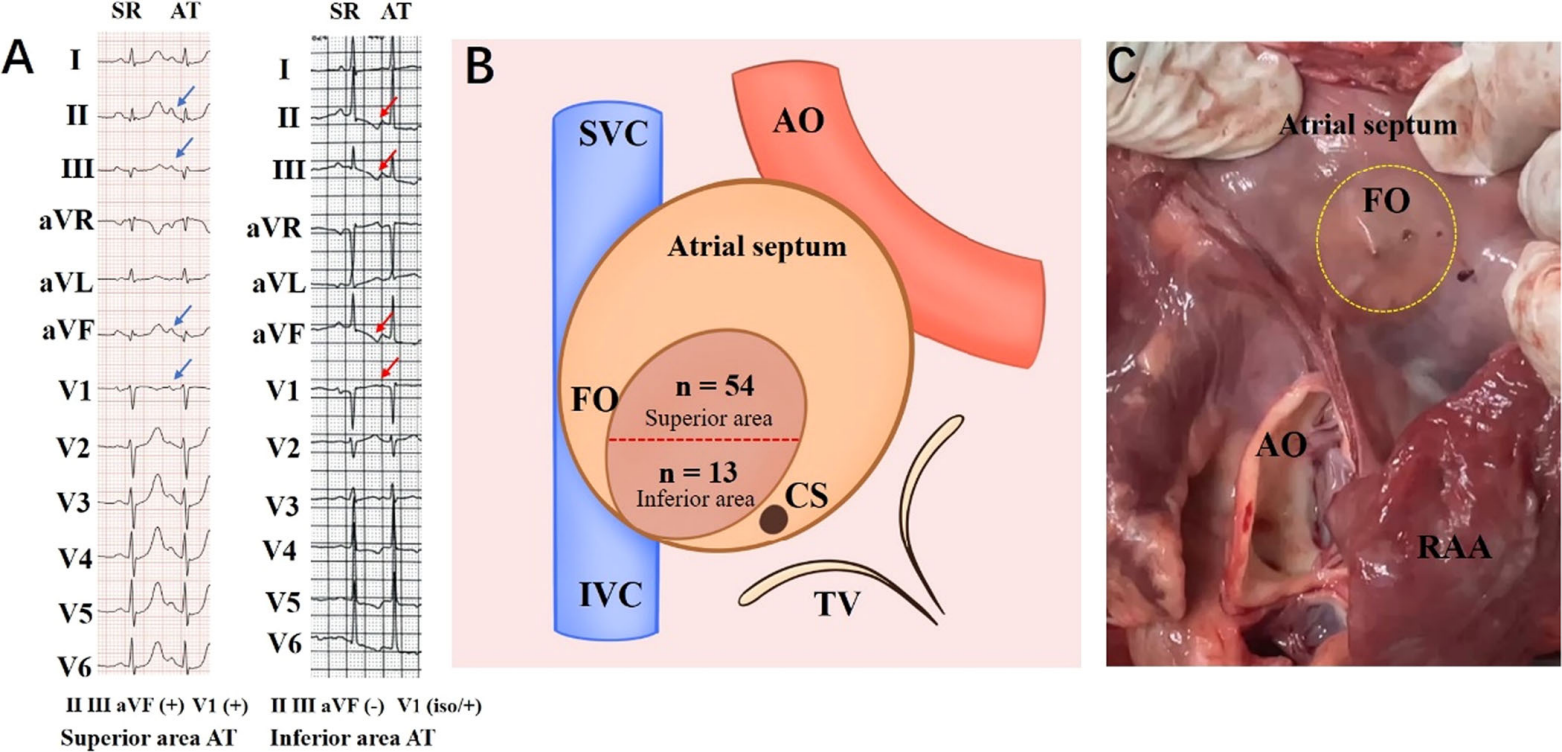
P‐wave morphology of AT originating from FO, diagrammatic representation of FO, and features of FO in a pig heart. (A) Representative 12‐lead electrocardiograms in two separate patients with FAT originating from the FO. (B) Diagrammatic representations of the FO and its anatomical relations. (C) Right atrial endocardial surface with a FO and its anatomical relations. The heart obtained from a pig. AO = Aorta, AT = atrial tachycardia, CS = coronary sinus, FO = fossa ovalis, IVC = inferior vena cava, RAA = right atrial appendage, SR = sinus rate, SVC = superior vena cava.

The PWM of FAT for the FO is depicted in Figure 2. Generally, the present study showed that a negative (*n* = 19) or biphasic (+/‐) (*n* = 30) P wave in lead V1 favored a r right earliest FO AT, whereas a positive (*n* = 16) or biphasic (‐/+) (*n* = 2) P wave in lead V1 favored a left earliest FO AT. The initial deflection of the P‐wave was typically positive (*n* = 54) and negative (*n* = 13) in leads II, III, and aVF. Based on the PWM in the inferior leads, patients were divided into two areas: the superior and inferior areas of the FO (Table 2).

**Table 2:** Procedural Characteristics of FAT originating from FO between two groups.

Variables	Unilateral ablation group (*n* = 36)	Bilateral ablation group (*n* = 31)	*p*
Total procedure duration, min	133.3 ± 13.4	152.4 ± 15.4	< 0.001
Fluoroscopic exposure time, min	14.6 ± 6.1	19.8 ± 7.7	0.006
RF time, min	11.9 ± 5.3	26.8 ± 7.8	< 0.001
Acute procedural success	36 (100%)	31 (100%)	1.000
Recurrence, n (%)	6 (16.6%)	0 (0%)	0.026
FO thickness at target	1.88 ± 0.50	1.86 ± 0.69	0.903

*Note:* Values are mean ± SD or *n* (%).

Abbreviations: FO = fossa ovalis, RF = radiofrequency.

### 3.4 Procedural Details

**Figure 3:**
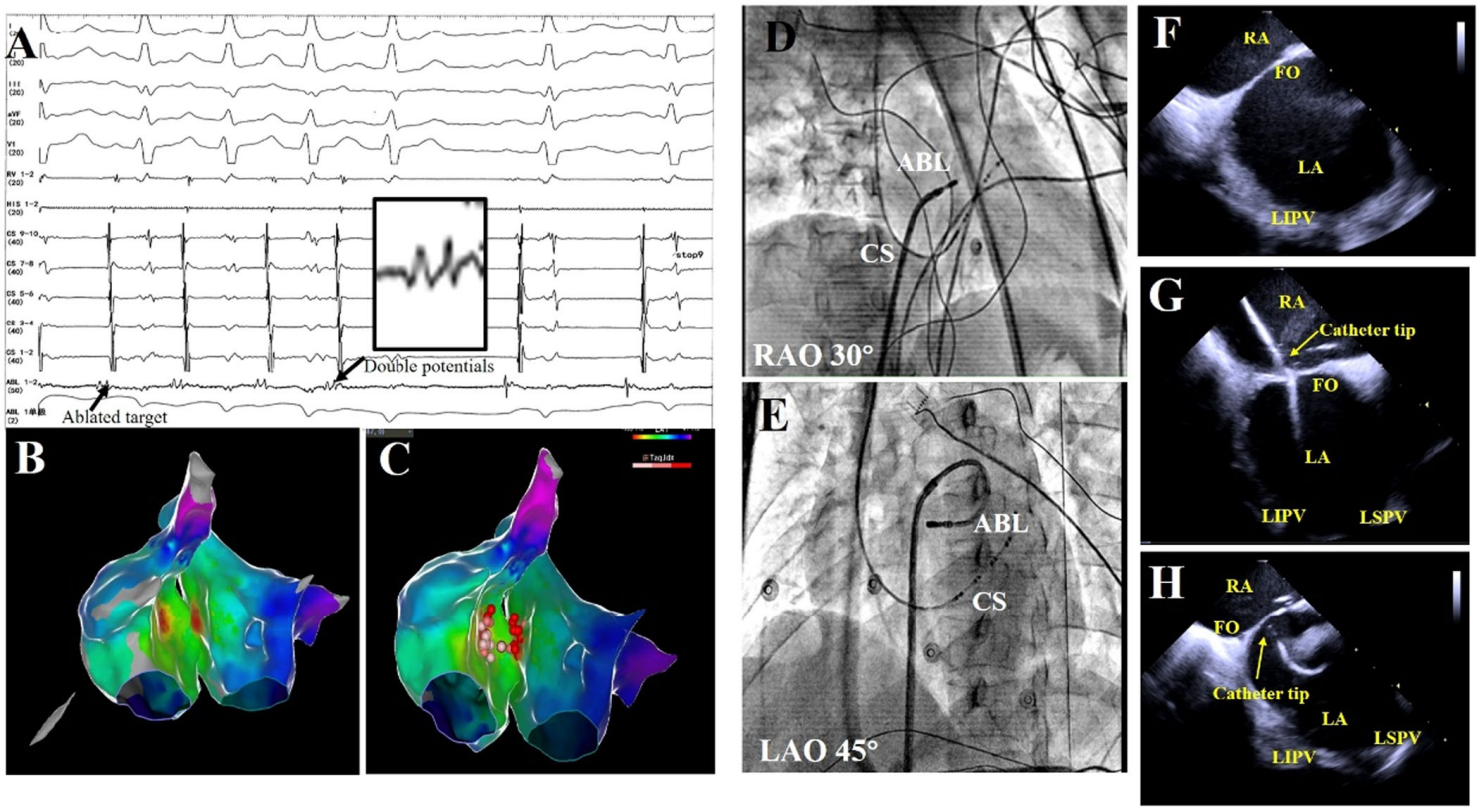
The ablation targets for a patient with FAT originating from FO in the Bilateral Ablation Group. (A) Cycle lengths of FAT in intracardiac electrogram and the ablation catheter on the right‐sided FO with double potentials. (B) Three‐dimensional electroanatomic map showing the ablated target of FAT in this group. The earliest atrial activation was recorded at the right and left atrium of FO. (C) Ablation performed at both the right and left earliest sites. (D) Fluoroscopic view of the successful right‐sided ablation site of FO at the left anterior oblique 45°. (E) Fluoroscopic view of the successful left‐sided ablation site of FO at the left anterior oblique 45°. (F) Intracardiac ultrasound image showing the FO. (G) Intracardiac ultrasound demonstrating the catheter tip contact with the right‐sided FO. (H) Intracardiac ultrasound demonstrating the catheter tip contact with the left‐sided FO.

**Figure 4:**
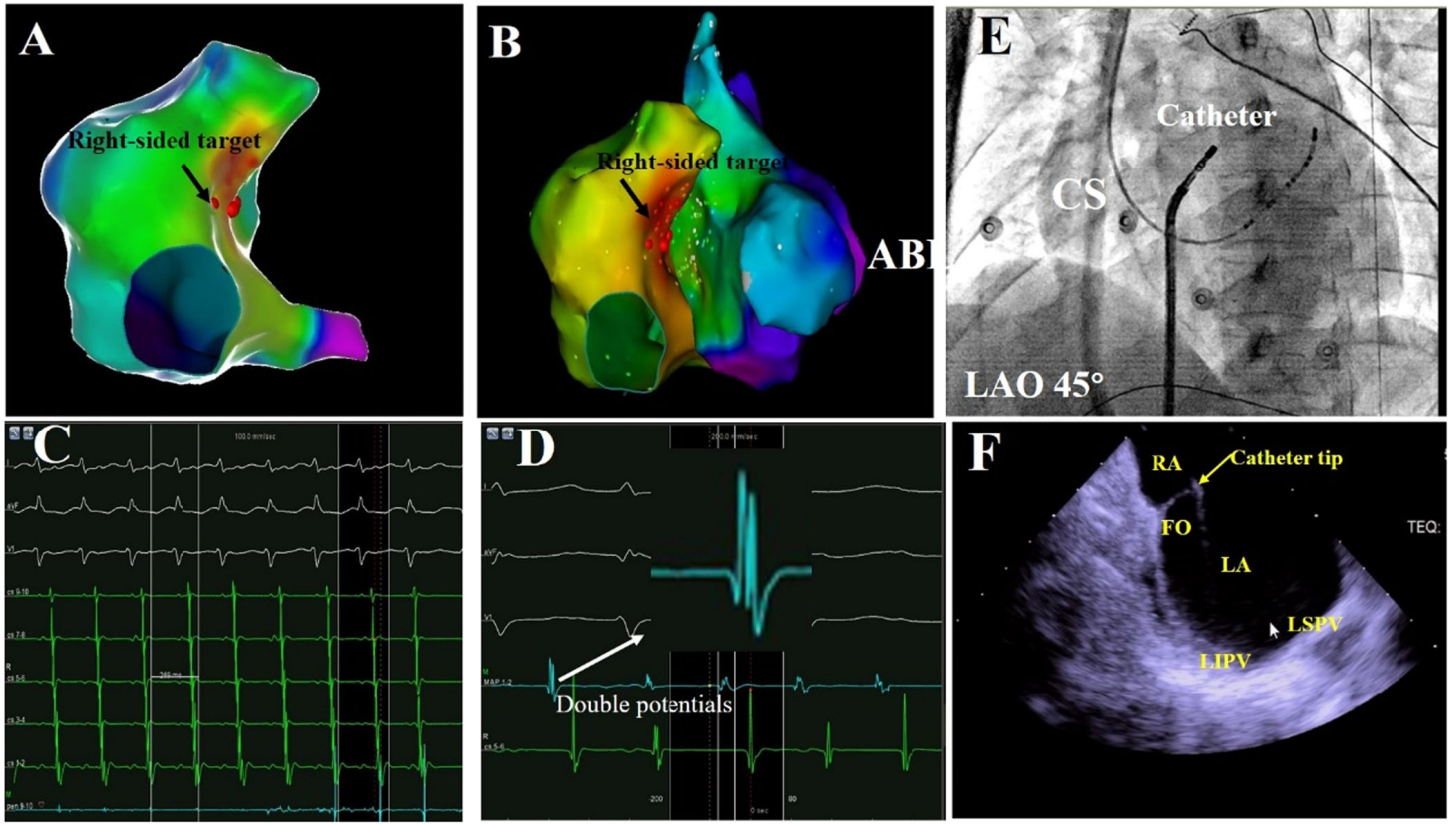
The ablation targets for a patient with FAT originating from the right‐sided FO in the Unilateral Ablation Group. (A, B) They depict the three‐dimensional electroanatomic map and the ablated target of FAT in this group. The earliest atrial activation was recorded at the right atrium of FO. (C) It provides a fluoroscopic view of the successful right‐sided ablation site of FO at the left anterior oblique 45°. (D, E) It displays the cycle lengths of FAT in intracardiac electrogram and the ablation catheter on FO with double potentials. (F) It shows intracardiac ultrasound directly demonstrating the catheter tip contact with FO.

**Figure 5:**
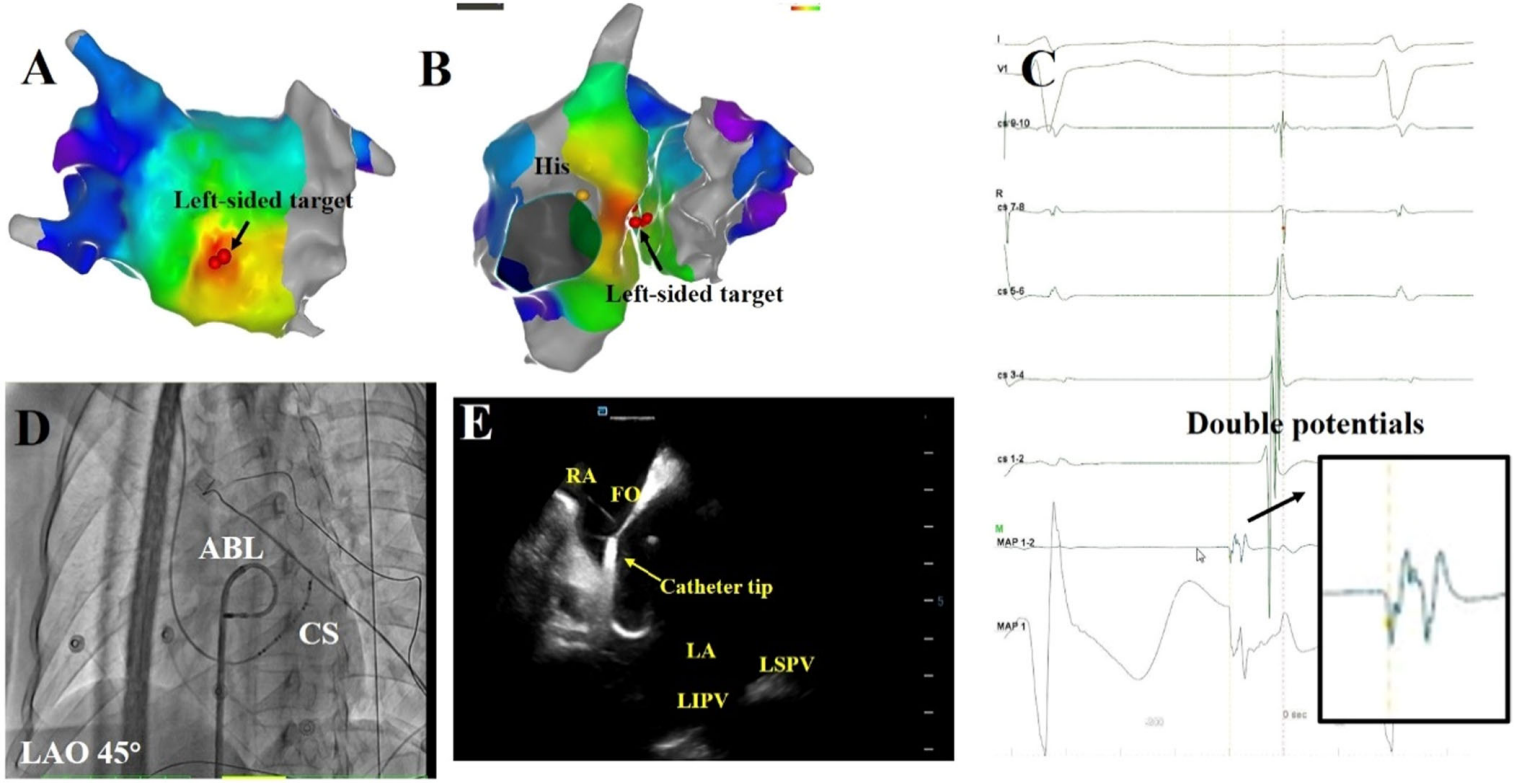
The ablation targets for a patient with FAT originating from the left‐sided FO in the Unilateral Ablation Group. (A, B) They depict the three‐dimensional electroanatomic map and the ablated target of FAT in this group. The earliest atrial activation was recorded at the left atrium of FO. (C) It displays the intracardiac electrogram and the ablation catheter on FO with double potentials. (D) It provides a fluoroscopic view of the successful left‐sided ablation site of FO at the left anterior oblique 45° by a reversed *U*‐curve catheter method. (E) It shows intracardiac ultrasound directly demonstrating the catheter tip contact with FO.

**Figure 6:**
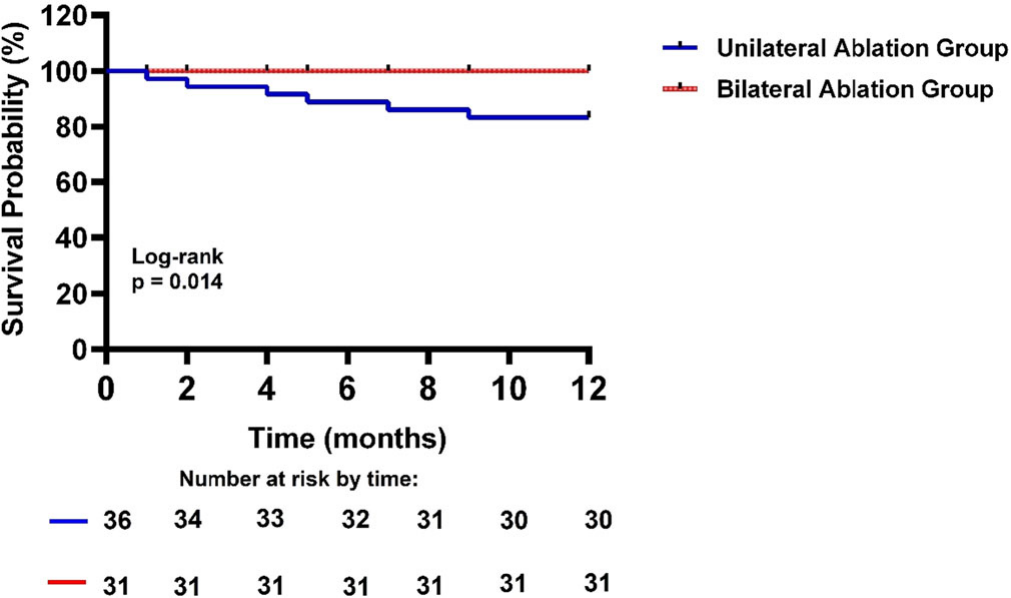
Kaplan−Meier survival curve from focal AT recurrence between two groups during the follow‐up.

The ablation details for a patient in the Bilateral group were presented in Figure 3, whereas the ablation details for two patients in the Unilateral Ablation Group were shown in Figures 4 and 5. All patients underwent successful ablation under the Carto system or Ensite system. The Bilateral Ablation Group had significantly longer total procedure duration, fluoroscopic exposure time, and RF time compared to the Unilateral Ablation Group (all *p* < 0.05). Both groups had a 100% acute procedural success rate. During a follow‐up at a mean of 26 ± 7 months without antiarrhythmic drugs, the patients in Unilateral Ablation Group experienced a significantly higher recurrence rate compared to that in the Bilateral Ablation Group, which had no recurrences (*p* = 0.026). The Kaplan−Meier survival curve revealed significant difference in focal AT recurrence between the two groups (log‐rank *p* = 0.014) (Figure 6). There were six patients with recurrences in the Unilateral Ablation Group, occurring at a mean time of 5 ± 3 months after ablation. In contrast, the Bilateral Ablation Group had no recurrences. Six recurrent cases were all treated by bilateral ablation method. Follow‐up showed no further recurrence. There were no complications during the procedure. During the follow‐up, patients were not on antiarrhythmic medications. Additionally, there was no significant difference in the FO thickness at the target site between Unilateral Ablation Group and Bilateral Ablation Group (1.88 ± 0.50 mm vs. 1.86 ± 0.69 mm, *p* = 0.903).

### 3.5 Procedural Details Comparison Between Recurrent and Non‐Recurrent Patients in the Unilateral Ablation Group

The procedural details for patients in the Unilateral Ablation Group were compared between those with recurrence (*n* = 6) and those without recurrence (*n* = 30) as shown in Table 3. There was no significant difference in the maximum Ablation Index (AI) between patients with and without recurrence (520 ± 57 vs. 514 ± 64, *p* = 0.945). The mean AI was also not significantly different between the two groups (414 ± 38 vs. 394 ± 68, *p* = 0.598). Additionally, there were no significant differences in minimum AI (280 (229−376) vs. 297 (233−372), *p* = 0.885), RF time (8 [6−9] min vs. 7 [5−9] min, *p* = 0.826), and the number of RF pulses (11 [8−15] vs. 10 [7−12], *p* = 0.763) between patients with and without recurrence. The mean contact force was similar between the recurrent and non‐recurrent groups (9.3 ± 2.9 g vs. 8.9 ± 2.6 g, *p* = 0.619), indicating no significant difference. Additionally, there was no significant difference in the FO thickness at the target site between patients with and without recurrence (1.98 ± 0.46 mm vs. 1.85 ± 0.51 mm, *p* = 0.664). Overall, the comparison of procedural details between patients with and without recurrence in the Unilateral Ablation Group revealed no significant differences in the measured variables, suggesting that these procedural factors were not associated with recurrence in this cohort.

**Table 3:** Comparison of parameters between patients with and without recurrence in the Unilateral Ablation Group.

Variables	Patients with recurrence (*n* = 6)	Patients without recurrence (*n* = 30)	*p*
Maximum AI	520 ± 57	514 ± 64	0.945
Mean AI	414 ± 38	394 ± 68	0.598
Minimum AI	280 (229−376)	297 (233−372)	0.885
RF time (min)	8 (6−9)	7 (5−9)	0.826
Number of RF pulses	11 (8−15)	10 (7−12)	0.763
Mean contact force (g)	9.3 ± 2.9	8.9 ± 2.6	0.619
FO thickness at target (mm)	1.98 ± 0.46	1.85 ± 0.51	0.664

*Note:* Values are mean ± SD, or median (1st–3rd quartile).

Abbreviations: AI = Ablation Index, FO = fossa ovalis, RF = radiofrequency.

## 4 Discussion

### 4.1 Major Findings

The major findings in this study are as follows: (1) Approximately 3.5% of patients with AT had FAT originating from the FO. (2) Patients with FAT originating from FO exhibited specific electrocardiographic characteristics, with 54 patients showing positive P waves in inferior leads and 13 patients showing negative P waves in inferior leads. (3) The bilateral ablation had lower recurrence rate than unilateral ablation.

Focal AT tends to originate from specific anatomical locations rather than occurring randomly throughout the atria. In the RA, these locations include the crista terminalis, tricuspid annulus, CS ostium, right‐sided septum, and perinodal area. In the LA, sites include pulmonary vein ostia, mitral annulus, LA appendage, and left‐sided septum [8]. Focal AT rarely originates from the interatrial septum (IAS). Chen et al. first described the electrophysiologic characteristics of right septal AT, with approximately 7.1% of tachycardias originating from the IAS in their study of 141 focal AT cases [9]. In our study, the largest of its kind, we analyzed data from 1914 patients with focal AT. Among them, 129 of 1914 (6.7%) were located in the right IAS, while 29 of 1914 (1.5%) were in the left IAS.

The IAS separates the left and right atria of the heart and is composed of several components, including the septum primum, septum secundum, and FO. The FO is a small depression in the interatrial septum, located in the region where the septum primum and septum secundum meet [10, 11, 12]. During fetal development, the FO represents the site of the foramen ovale, a shunt that allows blood to bypass the lungs. After birth, the FO normally closes, leaving behind the FO as a remnant. No previous analysis has examined the proportion of AT originating from the FO. Our study represents the first largest study to analyze the proportion of FO AT relative to all atrial tachycardias. In our study, we observed approximately 3.5% of patients with AT had FAT originating from the FO. The FO is a complex anatomical structure characterized by variations in tissue thickness and fibrous bands. These anatomical variations can create areas of slow conduction or areas with different conduction properties, leading to the generation of double potentials [5, 13]. In our study, we observed that patients with FO AT exhibited double potentials in the suitable ablation targets. Patients with FO AT exhibited double potentials at successful ablation sites. While the double potential's baseline configuration in SR primarily represents anatomical signal overlap between the left atrium (far‐field) and right septum (near‐field), its morphology during AT consistently showed dynamic changes (increased sharpening and widening). These morphological alterations signify functionally delayed conduction or local block due to anisotropic tissue properties or microreentry within the FO region, irrespective of the underlying anatomy. Thus, the presence of a double potential—particularly with tachycardia‐specific morphological changes—serves as an electrophysiological marker identifying sites suitable for ablation. The presence of double potentials serves as an important electrophysiological marker during ablation procedures, helping to identify specific sites within the FO that are suitable targets for intervention.

Given a predefined distribution, the P‐wave on surface 12‐lead ECG may provide a useful guide in determining the likely area of origin [14, 15]. The P‐wave morphology in lead V1 can vary based on the underlying cause of the arrhythmia, including the site of origin (e.g., left‐sided or right‐sided FO) and the direction of atrial depolarization. The current study revealed that a negative P wave (*n* = 19) or biphasic P wave with negative component (*n* = 30) in lead V1 favored a right earliest FO AT, while a positive P wave (*n* = 16) or biphasic P wave with positive component (*n* = 2) in lead V1 favored a left earliest FO AT. Based on the PWM in the inferior leads (II, III, and aVF), the study divided the FO into two areas: the superior area (a positive PWM in inferior leads) and the inferior area (a negative PWM in inferior leads). This division allows for a more precise localization of the origin of FAT within the FO based on the PWM in the inferior leads. It helps in characterizing the electrocardiographic features associated with FAT originating from different regions of the FO, which can guide the selection of appropriate ablation strategies and improve procedural outcomes.

**Figure 7:**
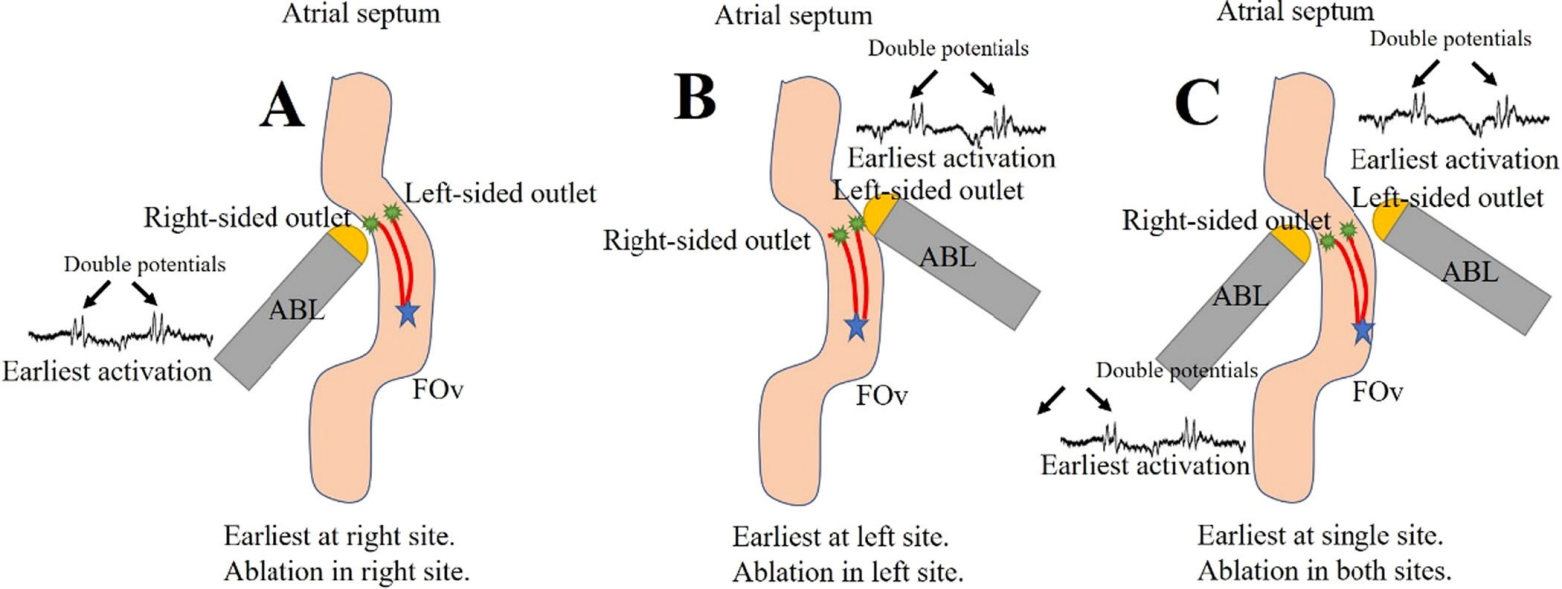
Catheter ablation of FAT originating from the FOv. (A) The right‐sided ablation of FAT was performed due to the earliest atrial activation at the right side of FOv. (B) The left‐sided ablation of FAT was performed due to the earliest atrial activation at the left side of FOv. (C) The earliest atrial activation located at one side of FOv, but both‐sided ablations of AT were performed. FAT = focal atrial tachycardia; FOv = fossa ovalis.

Medical therapy often proves ineffective in patients with FAT, prompting the utilization of catheter ablation as a safe and efficient treatment targeting the tachycardia focus [16, 17]. Catheter ablation has emerged as the preferred therapy for symptomatic FAT. However, while the FO is rarely the primary site of AT origin, it may contain arrhythmogenic foci that pose challenges for ablation. This difficulty may stem from the intricate anatomy of the FO, characterized by a raised rim and a deep annular recess formed during embryological development [18]. Previous research has suggested that bilateral mapping and ablation could be more effective. Indeed, a study found that the earliest RA activation at the mirror position of the FO coincided with the earliest LA activation [7]. Consequently, bilateral ablation may be more advantageous. The results indicate that bilateral ablation in the FO is significantly more effective than unilateral ablation. The effectiveness of bilateral ablation can be attributed to the more extensive ablation applied, which likely addresses a broader area of the arrhythmogenic substrate (Figure 7). This comprehensive approach ablates the opposite arrhythmic breakout that could lead to recurrence. The data clearly demonstrate that patients undergoing bilateral ablation have a more favorable outcome. Our current study supports this notion, as we observed a lower recurrence rate with bilateral ablation compared to unilateral approaches. Thus, bi‐atrial mapping is deemed necessary for the successful ablation of FAT originating from the FO.

### 4.2 Limitations

The limitations of this study include: (1) The study sample comprised 67 patients, which may limit the generalizability of the findings. A larger sample size would enhance the robustness of the results and allow for better evaluation of outcomes, especially considering the relatively low prevalence of FAT originating from the FO. (2) Although all patients were followed up for more than 1‐year, longer‐term follow‐up data would be valuable to assess the durability of ablation outcomes and the recurrence rate of atrial tachycardia over an extended period.

## 5 Conclusion

Bilateral ablation targeting both the right and left earliest sides of the FO appears to be a more rational approach, as it demonstrated a lower recurrence rate compared to unilateral ablation.

## Ethics Statement

The studies involving human participants were reviewed and approved by The Second Xiangya Hospital of Central South University, Wuhan Renmin Hospital of Wuhan University, Xiangyang Central Hospital, and Huichang County People's Hospital. The patients/participants provided their written informed consent to participate in this study. Written informed consent was obtained from the individual(s) for the publication of any potentially identifiable images or data included in this article.

## Conflicts of Interest

The authors declare no conflicts of interest.

## Data Availability

The datasets used and/or analyzed during the current study are available from the corresponding author on reasonable request.
